# A meta-analysis of the incidence of acne vulgaris in patients treated with GLP-1 agonists

**DOI:** 10.1097/JW9.0000000000000143

**Published:** 2024-04-05

**Authors:** Oluwafunke O. Ogunremi, Sana F. Ismail, Ramneek K. Dhami, Jazmin S. Newton, Scott A. Kindle, Valeriy Kozmenko

**Affiliations:** a Department of Internal Medicine, University of South Dakota Sanford School of Medicine, Vermillion, South Dakota; b Kansas City University College of Osteopathic Medicine, Kansas City, Missouri; c University of Nevada, Reno School of Medicine, Reno, Nevada

**Keywords:** acne vulgaris, GLP-1 agonists, glucagon-like-peptide-1 receptor agonists, liraglutide, Ozempic, semaglutide

## Abstract

**Background:**

With the emerging popularity of GLP-1 receptor agonists, patients are noticing acne vulgaris side effects that are seemingly related to the concurrent treatment with the drug. Due to the correspondence between these drugs’ relatively recent emergence in the U.S. market and their high demand, it is important to investigate what is currently known in the literature so that patients can be properly informed.

**Objective:**

The aim of this study is to investigate the relationship, or lack thereof, between glucagon like peptide 1 (GLP-1) receptor agonist usage and acne-related side effects in patients.

**Methods:**

A web-based analysis of 6 GLP-1 receptor agonists (3 with a once-weekly dosing schedule, and 3 with a once-daily dosing schedule) was conducted on PubMed online database. Boolean criteria were used to narrow the search. Included in the meta-analysis were 45 research articles that fulfilled the search criteria.

**Results:**

The results of the search showed that from the following long-acting GLP-1 receptor agonists, dulaglutide, exenatide extended release, and semaglutide (Wegovy), no conclusive acne side effects were reported. In addition, the results also showed that from the following short-acting GLP-1 receptor agonists, liraglutide, lixisenatide, and semaglutide (Rybelsus), no conclusive acne side effects were reported.

**Limitations:**

Limitations of this study include a limited amount of literature regarding the relationship between GLP-1 agonists and acne vulgaris.

**Conclusion:**

It is unlikely that GLP-1 agonists themselves are directly responsible for the acne that some patients may develop during treatment. Rather, it is more probable that the weight loss yielded by treatment with these drugs may induce intrinsic physiologic and hormonal changes that induce or exacerbate acne vulgaris in such patients.

**What is known about this subject in regard to women and their families?**

- Glucagon-like peptide 1 (GLP-1) agonists can be used in the treatment of women with polycystic ovary syndrome to help aid in weight loss as well as to reduce androgen levels. This treatment can reduce side effects such as excessive hair growth and acne that women with high levels of androgens may experience.

**What is new from this article as messages for women and their families?**

- This article helps inform women that using GLP-1 agonists in their treatment plan is not known to be linked to an increase in acne occurrence. This can help reassure women (as well as others) to keep to their treatment plan as per their doctor’s orders without fear of acne side effects that may adversely affect facial and bodily esthetics.

## Introduction

Blood sugar (also known as blood glucose) is the primary source of energy within the body. When blood sugar levels rise, insulin is released from the pancreas to lower blood sugar and increase the uptake of the sugar into cells. In homeostasis, normal blood sugar ranges from 70 to 100 mg/dL and depends on when the last meal was consumed.^1,2^ Specifically, fasting blood sugar should be less than 100 mg/dL. Diabetes mellitus (DM) is a disease state caused by the pancreas’ inability to produce insulin, as in type I DM, or the body’s inability to utilize the insulin produced, a so-called insulin sensitivity, as is the case in type II DM (T2DM). Those with a fasting blood sugar of 126 mg/dL or higher, or random blood sugar of greater than 200 mg/dL, are considered diabetic. In either form of DM, the body is unable to adequately regulate blood sugar levels leading to hyperglycemia, which can lead to a number of problems in the body, especially of the cardiovascular system.^1,2^ Specifically, T2DM is a chronic, often progressive disease that presents in mid-to-late adulthood and is a major cause of morbidity and mortality worldwide.^1–3^

There are a number of risk factors linked to the development of T2DM, one of the most significant is obesity.^2^ Obesity is defined as a body mass index >30 kg/m^2^.^2^ There is a strongly positive correlation between obesity and the development of T2DM, hence lifestyle choices such as maximizing diet and exercise are often the first recommendation for those with mild hyperglycemia (or prediabetes) to regain blood sugar control. However, medications are often necessary to help in these efforts.^1,2^ Metformin, which works primarily by lowering glucose production in the liver, is typically the first-line medication for those with T2DM.^1^ Given the complexity of blood sugar regulation at the individual level, many other medications acting by a number of different mechanisms may also be recommended depending on the patient. Glucagon-like peptide (GLP) is an endogenous hormone that acts on receptors in the pancreas to stimulate the production of insulin when blood sugar levels rise.^1,3^ It also decreases glucagon secretion. Additionally, GLP acts on receptors in the central nervous system and gastrointestinal tract, which delay gastric emptying, resulting in a reduction in appetite (increased satiety) and delayed glucose absorption.^1^ For those individuals who have insulin resistance (insensitivity) and/or are diagnosed with T2DM, GLP-1 receptor agonists are oral or injectable medications that mimic this natural hormone and may be recommended to help manage blood sugar in some patients.^1,3^

While GLP-1 agonists have traditionally been used as a second- or third-line treatment for T2DM, they have gained popularity among mainstream society given their weight loss side effects. Although the most common side effects, nausea and constipation, are viewed as negative consequences of taking this medication, many people consider weight loss to be a positive side effect. Studies have shown that GLP-1 agonists have promising potential to act solely as weight loss medications in those with or without T2DM, due to their ability to slow gastric emptying and increase satiety.^1,4–6^ While at one time these medications were used off-label as weight loss medications, the compelling clinical trial evidence led to recent Food Drug Administration approval of these drugs for the purpose of chronic weight management in those who are overweight or obese.^5,6^ Evidence supports the use of the GLP-1 agonists semaglutide and liraglutide for this purpose.

Not surprisingly, the uptick in the use of GLP-1 agonists for weight loss has given rise to increased discussion of their side effects, including those that may impact body image and aesthetics. For example, one recent concern included the potential link between GLP-1 agonists (specifically semaglutide) and hair loss. However, this suspicion was not confirmed by other studies.^7,8^ Along those same lines, the development of acne vulgaris in relation to starting GLP-1 agonists is now being questioned. Acne vulgaris is caused by excessive sebum production, inflammation, follicular hyperkeratinization, or Propionibacterium acnes bacterial colonization of the pilosebaceous unit in the skin.^9^

Multiple factors have been shown to contribute to acne development, including genetic, hormonal, inflammatory, and environmental influences.^9,10^ Hormones modulate the pilosebaceous unit, especially androgens.^9^ Androgens promote an increase in the size of sebaceous glands in the skin, which increases the amount of sebum (oil) produced, which can lead to clogged pores and acne.^9,10^ While acne can occur at any age, it is most common in adolescence due to the hormonal changes that occur during puberty.^10^ Hormones can also contribute to the development of adult acne, especially in women who may be pregnant, perimenopausal, or changing birth control methods. Environmental factors that contribute to acne include but are not limited to nicotine use, diet, cosmetic products, stress, and conditions that impact metabolism such as diabetes or obesity. Adipose tissue can cause an inflammatory response, as in the case of visceral adipose tissue.^11–13^ Obesity in the form of visceral adipose increases the blood glucose level and contributes to insulin resistance, which can influence the level of circulating androgens and hence acne development.^9–13^ Adipose tissue itself also contains both estrogen and androgen receptors.^9,12,13^ Studies have shown that patients with acne who consume food that has a lower glycemic load tend to have less lesions than those who consume diets with a higher glycemic load.^10^ Weight loss has been shown to improve factors that contribute to both the inflammatory and endogenous factors that contribute to acne.^13^ Thus, in the case of GLP-1 agonist users, it is suspected that acne would actually improve with their use because they help regulate metabolic and hormonal abnormalities related to blood glucose, insulin resistance, and obesity.

While studies regarding hair loss have dispelled the myth that GLP-1 agonists were a direct cause of nonscarring hair loss, little work has been done to examine the possible relationship between GLP-1 agonists and acne vulgaris. Here, we performed a meta-analysis of the present literature, discussed key findings, and offered recommendations for both patients and clinicians.

## Methods

**Fig. 1.**
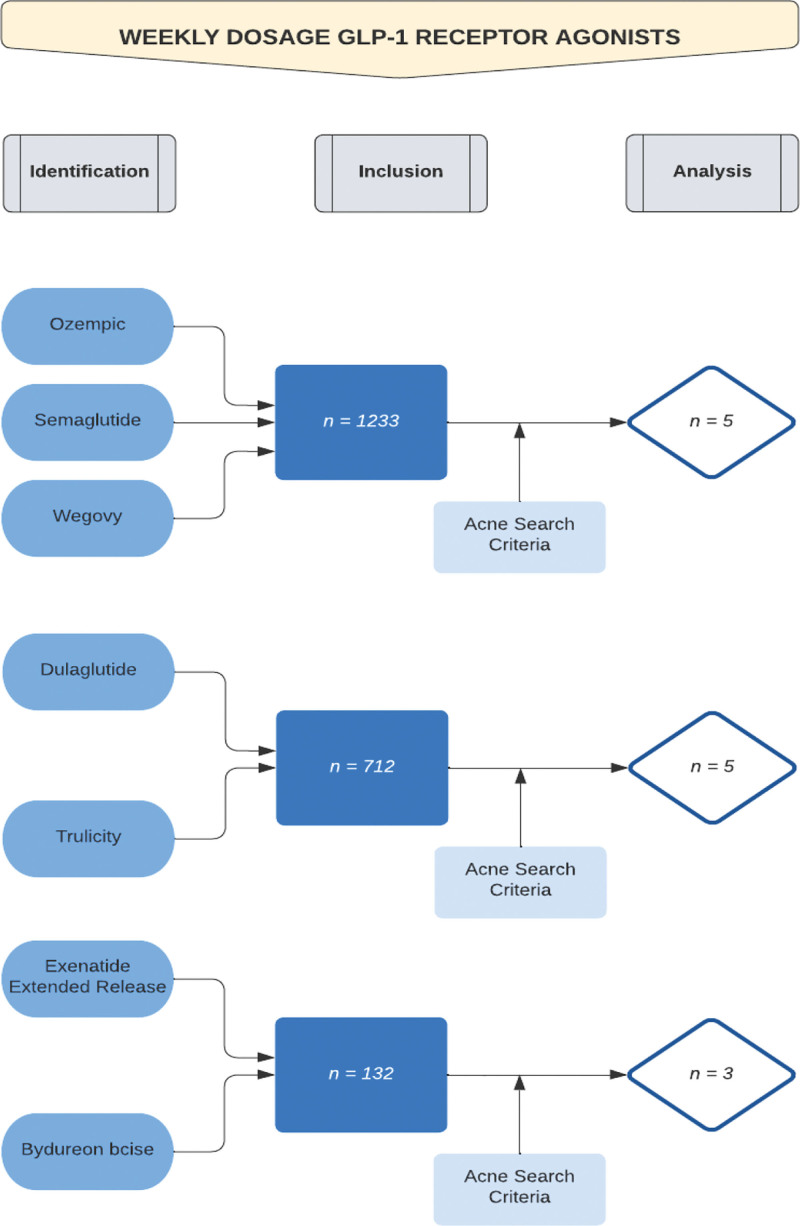
PubMed search of weekly dosage glucagon-like peptide 1 (GLP-1) receptor agonists. The process utilized to narrow the PubMed search for the identification of research articles analyzed for the weekly dosage GLP-1 receptor agonists is depicted. The Boolean method was utilized to achieve the final results. For Ozempic/semaglutide/Wegovy, 1223 research articles were found prior to inclusion of the acne search key terms. Following the addition of the acne key terms to the search, 5 articles were found to be valid for analysis of Ozempic/semaglutide/Wegovy. For dulaglutide/Trulicity, 712 research articles were found prior to inclusion of the acne search key terms. Following the addition of the acne key terms to the search, 5 articles were found to be valid for analysis of dulaglutide/Trulicity. For exenatide extended release/Bydureon BCise, 132 research articles were found prior to inclusion of the acne search key terms. Following the addition of the acne key terms to the search, 3 articles were found to be valid for analysis of exenatide extended release/Bydureon BCise.

**Fig. 2.**
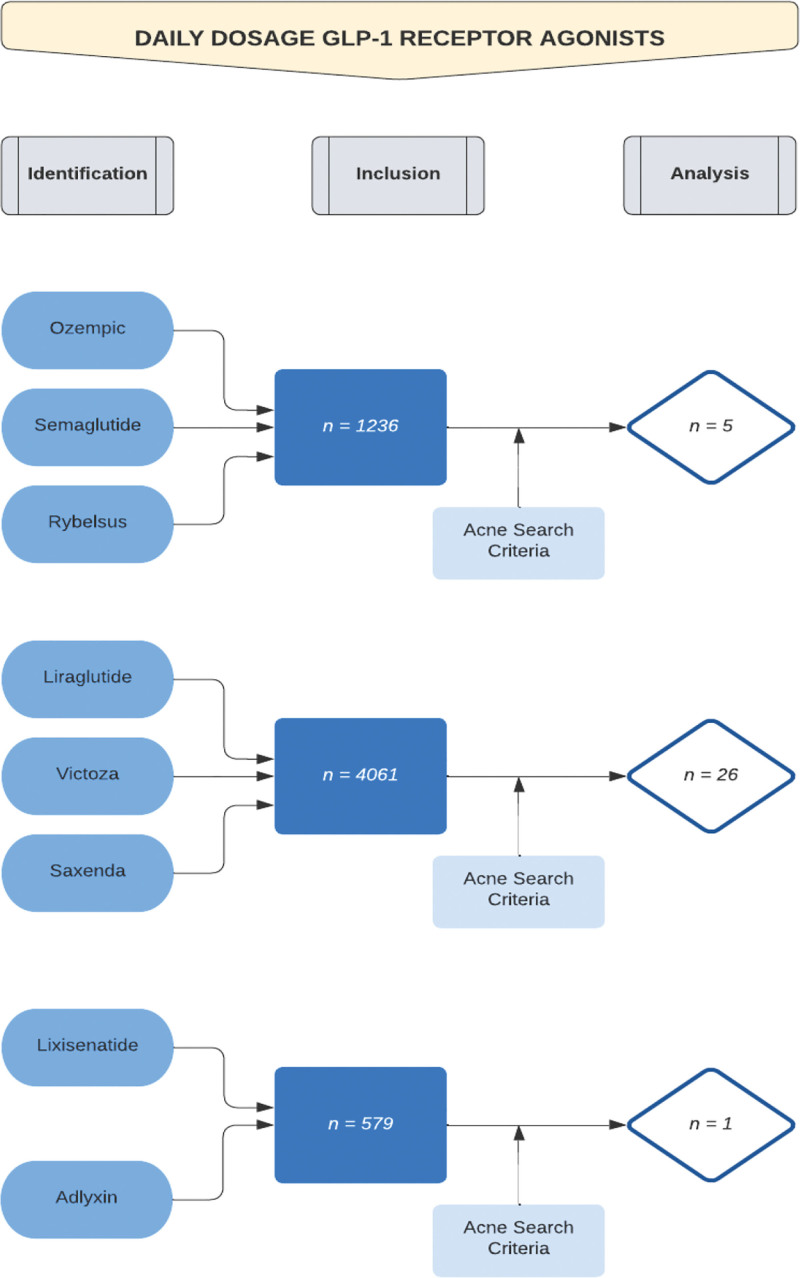
PubMed search of daily dosage glucagon-like peptide 1 (GLP-1) receptor agonists. The process utilized to narrow the PubMed search for the identification of research articles analyzed for the daily dosage GLP-1 receptor agonists is depicted. The Boolean method was utilized to achieve the final results. For Ozempic/semaglutide/Rybelsus, 1236 research articles were found prior to inclusion of the acne search key terms. Following the addition of the acne key terms to the search, 5 articles were found to be valid for analysis of Ozempic/semaglutide/Rybelsus. For liraglutide/Victoza/Saxenda, 4061 research articles were found prior to inclusion of the acne search key terms. Following the addition of the acne key terms to the search, 26 articles were found to be valid for analysis of liraglutide/Victoza/Saxenda. For lixisenatide/Adlyxin, 579 research articles were found prior to inclusion of the acne search key terms. Following the addition of the acne key terms to the search, 1 article was found to be valid for analysis of lixisenatide/Adlyxin.

A web-based search was performed on 6 GLP-1 receptor agonists via the PubMed online database. Three of the chosen GLP-1 agonists were long acting and 3 were short acting. The purpose of this criteria was to analyze if a difference existed in terms of acne-related side effects caused by a difference in duration of action of the GLP-1 receptor agonists on the body. The Boolean method was used to help create search criteria. The long-acting agents, dulaglutide, exenatide extended release, and semaglutide (Wegovy), utilized a weekly dosing period (Fig. 1). The short-acting agents, liraglutide, lixisenatide, and semaglutide (Rybelsus), utilized a once-daily dosage (Fig. 2). In terms of the PubMed drug search criteria, the generic names and the brand drug names were used for the Boolean criteria. In terms of search criteria for associated acne side effects, the following keywords and phrases were chosen: acne, pimple, pustule, papule, nodule, cyst, zit, blackhead, and whitehead (Figs. 1 and 2). The search results are described in Table 1. Each article was then analyzed for the following pieces of information: number of patients involved in the study, documented acne-related side effects, and the sex of patients affected by the side effect.

**Table 1:** Search results for GLP-1 agonist medications

GLP-1 receptor agonist	Number of viable published research articles based on Boolean criteria
Dulaglutide (Trulicity)	5
Exenatide extended release (Bydureon BCise)	3
Semaglutide (Wegovy)	5
Semaglutide (Rybelsus)	5
Liraglutide (Victoza, Saxenda)	26
Lixisenatide (Adlyxin)	1

The final results achieved following the completion of the Boolean search method in the PubMed database are depicted. Thirteen total articles were analyzed for the weekly dosage glucagon-like peptide 1 (GLP-1) receptor agonists (dulaglutide [Trulicity]; semaglutide [Wegovy]; exenatide extended release [Bydureon BCise]). Thirty-two total articles were analyzed for the daily dosage GLP-1 receptor agonists (semaglutide [Rybelsus]; liraglutide [Victoza, Saxenda]; lixisenatide [Adlyxin]).

## Results

A total of 45 articles were analyzed based on the intersection of the 2 criteria groups. From these 45 articles, only 3 had findings consistent with a GLP-1 agonist triggering a side effect from the acne search criteria such as a nodule or cyst. Moreover, the drug associated with these 3 articles was the GLP-1 agonist exenatide extended release. The first article was a case report in which a 63-year-old woman presented with nodules on her abdomen.^14^ These nodules were found to be caused by exenatide-induced granulomatous panniculitis. The second article was also a case report and found nodule development on the thigh of a 54-year-old man. This was found to be exenatide-induced eosinophilic panniculitis.^15^ The third article included 27 reports from the Food Drug Administration Adverse Event Reporting System. Each one of the 27 cases reported one or more nodules related to an injection-site reaction.^16^

One article also analyzed the exenatide immediate release. Although the medication caused localized and nonserious reactions, there were no symptoms such as large nodules or indurations. Furthermore, the prevalence of injection-site reactions in relation to the extended release is considerably higher than for the immediate-release exenatide (17.1% vs 12.7%). These findings suggest the involvement of another agent in the exenatide extended release, but this is not certain. The symptoms such as nodules, warmth, and pruritus and the presence of granulomas and eosinophils in the pathology are also indicative of a hypersensitivity reaction to the extended-release formula.^17^

## Discussion

Though 3 articles strongly discussed one of the selected GLP-1 agonists in relation to one of the acne search criteria terms, they did not fulfill the criteria for a diagnosis consistent with acne vulgaris. None of the 3 articles that reported nodules or cysts reported the symptoms on the face, upper arms, or upper back, areas commonly targeted by acne vulgaris. All 3 articles mentioned local or injection-site discoloration and nodules. Even in analyzing the exenatide immediate release, there were no symptoms reported that align with acne vulgaris.

Among the articles analyzed in this study, there is a lack of evidence in support of GLP-1 agonists causing acne-related side effects in patients. However, many patients are taking to social media platforms such as Reddit, Twitter, and TikTok to document and report that they are experiencing an onset of facial acne coinciding with the early stages of their treatment with GLP-1 agonists. For example, the Reddit forum for semaglutide (r/semaglutide) contains 39,364 members who share their unique experiences with the drug. When searching the key term “acne” in the forum, 40 main posts are retrieved with 982 total comments between these posts. There seems to be a mixed review from the patients within the forum with some claiming that semaglutide has cleared their acne, while many others claim that it is responsible for the induction of new acne. Many of these people are attributing this symptom to the drug itself rather than considering underlying mechanisms. Patients treated with GLP-1 agonists often experience weight loss as a side effect of the drug. Moreover, weight loss may trigger a hormonal change in the body, which can induce or exacerbate acne vulgaris.^18^ An underlying mechanism related to acne vulgaris involves insulin-like growth factor 1 (IGF-1). The literature supports that an increase in weight loss leads to an increase in IGF-1.^19^ The results from one study suggest that IGF-1 may lead to the induction of acne or worsen preexisting acne through upregulating inflammatory biomarkers in sebaceous genes.^20^ After treating cultured sebocytes with IGF-1, a study performed polymerase chain reaction and enzyme-linked immunosorbent assay to evaluate changes in the expression of inflammatory markers such as interleukin (IL) 1β, IL-6, IL-8, tumor necrosis factor α, and nuclear factor kappa-light-chain-enhancer of activated B cells. They used lipid analysis to evaluate sebum production. They found that expression levels of the inflammatory markers, as well as gene expression levels of nuclear factor kappa-light-chain-enhancer of activated B cells, increased after IGF-1 treatment. Sebum production was also increased in the cultured sebocytes treated with IGF-1. This suggests that IGF-1 may play a role in the pathogenesis of acne vulgaris and understanding this underlying pathophysiology is important for patients to remain compliant in their medication management with GLP-1 agonists.^20^

Another explanation for the reactions caused by exenatide is that the injection-site nodules were not symptoms of acne vulgaris, but rather a hypersensitivity reaction. As aforementioned, nodules and pruritus and the presence of granulomas and eosinophils on histopathologic analysis suggest hypersensitivity reactions. This could be the result of an agent in the exenatide extended-release formula and would explain its exclusive reaction.

It is of importance to note that it may be that the GLP-1 agonists are reducing rather than inducing acne vulgaris. One study analyzed the circulating levels of GLP-1 and other incretins such as gastric inhibitory polypeptide in high-risk acne patients. The researchers analyzed 60 patients with acne vulgaris and 56 patients without acne vulgaris who served as the control group. All patients were between the ages of 13 and 40 years old. The results showed that there was a statistically significant lower average level of serum incretin in the acne vulgaris group compared to the control group. Furthermore, the results showed that among the patients with acne vulgaris, those with insulin resistance had statistically significant lower levels of incretin compared to the patients with acne vulgaris without insulin resistance. These results may indicate that serum incretin levels such as GLP-1 may potentially serve as a biomarker for the diagnosis of acne vulgaris in patients with insulin resistance.^21^ As one of the underlying mechanisms of GLP-1 agonists is to reduce insulin resistance by serving as an incretin mimetic, it is possible that the lack of findings in this literature meta-analysis in terms of GLP-1 agonists inducing acne vulgaris in patients is due to the fact that they are actually having the opposite effect, and instead reducing the incidence of acne vulgaris in patients with hyperinsulinemia by increasing serum incretin levels.

Limitations of this study include a limited amount of literature regarding the relationship between GLP-1 agonists and acne vulgaris. This is likely due to the fact that many of these drugs have become popular only very recently and studies specifically assessing dermatologic side effects (aside from commonplace injection-site reactions) have yet to be performed. However, future studies should analyze the dermatologic side effects of these medications as consumers share concerns regarding their impact on the skin.

## Conclusion

Overall, the present literature review revealed no direct correlation of acne vulgaris as a side effect of GLP-1 agonists. It is unlikely that GLP-1 agonists themselves are directly responsible for the acne that some patients may develop during treatment. Rather, it is more probable that the weight loss yielded by treatment with these drugs may induce intrinsic physiologic and hormonal changes that induce or exacerbate acne vulgaris in such patients. Nonetheless, further research is necessary to definitively characterize the nature of acne development in the setting of GLP-1 agonist use.

## Conflicts of interest

None.

## Funding

None.

## Study approval

N/A

## Author contributions

OOO, JSN: Participated in research design. OOO, SFI, RKD, JSN: Participated in the writing of the paper and performance of the research. OOO, SFI, RKD: Participated in data analysis. OOO, JSN, SAK, VK: Contributed by overseeing the research.
